# Safety, tolerability, and pharmacokinetics of VV116, an oral nucleoside analog against SARS-CoV-2, in Chinese healthy subjects

**DOI:** 10.1038/s41401-022-00895-6

**Published:** 2022-03-16

**Authors:** Hong-jie Qian, Yu Wang, Meng-qi Zhang, Yuan-chao Xie, Qing-qing Wu, Li-yu Liang, Ye Cao, Hua-qing Duan, Guang-hui Tian, Juan Ma, Zhuo-bing Zhang, Ning Li, Jing-ying Jia, Jing Zhang, Haji Akber Aisa, Jing-shan Shen, Chen Yu, Hua-liang Jiang, Wen-hong Zhang, Zhen Wang, Gang-yi Liu

**Affiliations:** 1 Central Laboratory, Shanghai Xuhui Central Hospital/Xuhui Hospital, Fudan University, Shanghai, 200031 China; 2 Shanghai Engineering Research Center of Phase I Clinical Research and Quality Consistency Evaluation for Drugs, Shanghai, 200031 China; 3 CAS Key Laboratory of Receptor Research, Drug Discovery and Design Center, Shanghai Institute of Materia Medica, Chinese Academy of Sciences, Shanghai, 201203 China; 4 Lingang Laboratory, Shanghai, 201602 China; 5 Clinical Department, Vigonvita Life Sciences Co., Ltd, Suzhou, 215123 China; 6 Research and Development Department, Shanghai Junshi Biosciences Co., Ltd, Shanghai, 200126 China; 7 Phase 1 Clinical Research Center, Huashan Hospital, Fudan University, Shanghai, 200040 China; 8 State Key Laboratory Basis of Xinjiang Indigenous Medicinal Plants Resource Utilization, Xinjiang Technical Institute of Physics and Chemistry, Chinese Academy of Sciences, Urumqi, 830011 China; 9 State Key Laboratory of Drug Research, Shanghai Institute of Materia Medica, Chinese Academy of Sciences, Shanghai, 201203 China; 10 Department of Infectious Diseases, Huashan Hospital, Fudan University, Shanghai, 200040 China

**Keywords:** VV116, nucleoside analog, SARS-CoV-2, safety, pharmacokinetics, healthy subjects

## Abstract

VV116 (JT001) is an oral drug candidate of nucleoside analog against SARS-CoV-2. The purpose of the three phase I studies was to evaluate the safety, tolerability, and pharmacokinetics of single and multiple ascending oral doses of VV116 in healthy subjects, as well as the effect of food on the pharmacokinetics and safety of VV116. Three studies were launched sequentially: Study 1 (single ascending-dose study, SAD), Study 2 (multiple ascending-dose study, MAD), and Study 3 (food-effect study, FE). A total of 86 healthy subjects were enrolled in the studies. VV116 tablets or placebo were administered per protocol requirements. Blood samples were collected at the scheduled time points for pharmacokinetic analysis. 116-N1, the metabolite of VV116, was detected in plasma and calculated for the PK parameters. In SAD, AUC and *C*_max_ increased in an approximately dose-proportional manner in the dose range of 25–800 mg. *T*_1/2_ was within 4.80–6.95 h. In MAD, the accumulation ratio for *C*_max_ and AUC indicated a slight accumulation upon repeated dosing of VV116. In FE, the standard meal had no effect on *C*_max_ and AUC of VV116. No serious adverse event occurred in the studies, and no subject withdrew from the studies due to adverse events. Thus, VV116 exhibited satisfactory safety and tolerability in healthy subjects, which supports the continued investigation of VV116 in patients with COVID-19.

## Introduction

The global pandemic of COVID-19 caused by the SARS-CoV-2 has been lasting for over 2 years and caused a significant impact on human life and human society. As the pandemic continues, how to return life to normal comes to be the top priority for the whole world. In the past year, remdesivir (RDV) [1], therapeutic neutralizing monoclonal antibodies [2, 3], and COVID-19 vaccines [4, 5] constituted the main measures for treating or preventing SARS-CoV-2 infection, saving the lives of many people who were infected or at high risk to be infected. In the end of 2021, two oral antivirals, nirmatrelvir/ritonavir [6, 7] and molnupiravir [8, 9], which are used to treat non-hospitalized patients diagnosed with mild to moderate COVID-19 who are at high risk for progression to severe COVID-19, received the FDA’s emergency use authorization. The effective small-molecule oral antiviral drugs with low cost, easy mass production, easy storage, and high accessibility for outpatients are considered as the game changer for COVID-19.

**Fig. 1:**
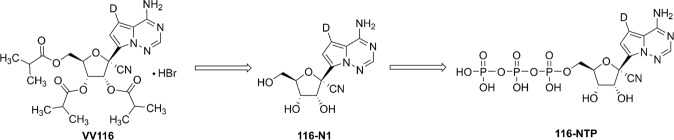
The structural formula of VV116, 116-N1 and 116-NTP. After oral administration, VV116 is rapidly converted to 116-N1, which undergoes three successive enzymatic phosphorylation reactions in cells to yield the triphosphate active form, 116-NTP.

Nucleoside analogs are one of the most important classes of antivirals that target the highly conserved active site of viral polymerase and function by being incorporated into new viral genomes to cause defective genomes [10, 11]. These kinds of antiviral drugs are characterized with high efficacy and low incidence of viral resistance [12, 13]. Recently, we reported a promising oral drug candidate VV116 (JT001) for treating SARS-CoV-2 infection by a comprehensive preclinical study [14]. VV116 is a deuterated, tri-isobutyrate ester prodrug of the RDV parent nucleoside, and is rapidly metabolized into the parent nucleoside (116-N1) in the body. 116-N1 is intracellularly converted to the nucleoside triphosphate active form, which would interfere with the function of RNA-dependent RNA polymerase of SARS-CoV-2, thus exerting antiviral effects (Fig. 1). VV116 showed potent activity against a panel of SARS-CoV-2 variants (alpha, beta, delta, and omicron) and excellent therapeutic efficacy in the mice model. This prodrug was endowed with significantly improved oral absorption and a favorable tissue distribution profile, circumventing the liver-targeting issue of the phosphoramidate prodrugs [14, 15].

VV116 has been approved for the treatment of COVID-19 in Uzbekistan based on the positive clinical trial results. The phase I clinical trial of VV116 in China has been completed and advanced to global phase II/III clinical trials (ClinicalTrials.gov Identifier: NCT05242042). Herein, we report the results of three phase I studies to evaluate the safety, tolerability, and pharmacokinetics of single and multiple ascending oral doses of VV116 in healthy subjects, as well as the effect of food on the pharmacokinetics and safety of VV116.

## Materials and methods

Three studies (Study 1, Study 2, and Study 3) were launched sequentially (Fig. 1). Study 1 and 2 were randomized, double-blind, placebo-controlled, single and multiple ascending-dose studies. Study 3 was a randomized, open-label, three-cycle, cross-over study to investigate the food effect of VV116.

The studies were accomplished at the Phase I Clinical Research Center of Shanghai Xuhui Central Hospital, Shanghai, China, from November 2021 to January 2022. The studies were conducted in accordance with the Declaration of Helsinki (World Medical Association) and Good Clinical Practice (GCP). The study protocol and informed consent forms were approved by the Ethics Committee of the Shanghai Xuhui Central Hospital. All of the subjects provided written informed consent before any study-related procedure was performed. The studies were registered on ClinicalTrials.gov (ClinicalTrials.gov Identifier: NCT05227768, NCT05201690, NCT05221138).

### Participants

Subjects who met the following inclusion criteria, while did not meet the exclusion criteria, were eligible to participate in the study.

Inclusion criteria:

- aged 18–45 years, regardless of sex;
- body weight≥50 kg for males, ≥45 kg for females, body mass index (BMI) of 19–26 kg/m^2^ (inclusive);
- normal vital signs, physical examination, 12-lead ECG, laboratory tests, ophthalmological examination and B-mode ultrasound examination, or abnormality with no clinical significance;
- willing to use effective contraceptive measures during the study and within 3 months after the last dose of investigational products.

Exclusion criteria:

- allergy history;
- history of any systemic disorders or diseases requiring medical intervention;
- blood donation or blood loss≥400 mL within 3 months, or history of blood product use;
- participation in any clinical trial within 3 months;
- any concomitant medication within 2 weeks prior to screening;
- drug or alcohol addicts, or heavy smokers within 1 year;

- positive test for hepatitis B, hepatitis C, HIV, or syphilis;
- pregnant or lactating female, or partners of male subjects who have a birth-giving plan within 3 months.

### Procedures

#### Study 1: single ascending-dose study

Based on the nonclinical toxicology data of VV116, the estimated maximum recommended starting dose was selected at 25 mg, which was equivalent to 1/100 and 1/48 of no observed adverse effect level (NOAEL) in the 14-day studies in rats and dogs, respectively. In the mean time, the estimated maximum dose was selected at 1200 mg. Initially, eight ascending-dose groups (25, 50, 100, 200, 400, 600, 800, and 1000 mg) were designed in the protocol. According to the safety results of the 25 mg dose group, as well as the ongoing clinical trial in Uzbekistan, adjustment was made for the dose group settings to 25, 200, 400, 800, and 1200 mg under the communication with Center for Drug Evaluation (CDE), NMPA, which was also approved by the Ethics Committee.

Eligible subjects were admitted to the Phase I Clinical Research Center 1 day before drug administration. On the morning of day 1, after overnight fasting for ≥10 h, subjects were administered VV116 tablet or placebo at the respective dose level with 240 mL water under fasting condition. All the subjects stayed at the Phase I Clinical Research Center until 48 h after dosing with close medical monitoring, except for subjects in 400-mg dose group, who stayed until 72 h after dosing for exploratory mass balance evaluation. A follow-up visit by telephone was performed by investigators on day 7 (±1 day).

#### Study 2: multiple ascending-dose study

Three ascending-dose groups (200, 400, and 600 mg) were designed in the protocol. Twelve subjects were enrolled in each dose group at a 3:1 ratio to receive the study drug or the placebo. Eligible subjects were admitted to the Phase I Clinical Research Center one day before drug administration. VV116 tablets or placebo were administered twice a day (12 h apart), continuous administration was given for 5.5 days (days 1–6). All the subjects stayed at the phase I unit until 48 h after the last dose. A follow-up visit by telephone was performed by investigators on day 12.

#### Study 3: food-effect study

In this part, 12 eligible subjects were randomized and equally assigned into three groups (Group A, B, and C). Each group experienced three treatment periods (fasting, fed with the standard meal, and fed with high-fat meal) with a 3-day washout period in between. For the fasting period, a single oral dose of 400 mg VV116 tablet was administered after an overnight fasting period (≥10 h). For the two fed periods, a single oral dose of 400 mg VV116 tablet was administered within 30 min after the consumption of a standard meal (total calories: approximately 700 kcal), or a high-fat meal (total calories: ~800–1000 kcal, which derives about 150, 250, and 500–600 kcal from protein, carbohydrate, and fat, respectively). All the subjects stayed at Phase I Clinical Research Center until 48 h after the last dose. A follow-up visit by telephone was performed by investigators on day 13.

### Safety assessment

Safety was assessed by vital signs, physical examinations, 12-lead ECG, clinical laboratory tests (hematology, blood chemistry, serum amylase and lipase, urinalysis, urinary sediment, urine microalbumin, urine *N*-acetyl-beta-*D*-glucosaminidase (NAG), coagulation function, thyroid function, and hormone test of reproductive system), thyroid B ultrasound, ophthalmology examination, and monitoring for adverse events (AEs) throughout the study. AEs were evaluated according to National Cancer Institute Common Terminology Criteria for Adverse Events (CTCAE, version 5.0). All AEs were coded using Medical Dictionary for Regulatory Activities (MedDRA, version 24.1).

The decision to proceed to the next dose cohort was made by both investigators and sponsor according to the dose-escalation termination criteria described in the protocol.

The dose-escalation termination criteria were determined by AEs. Dose escalation should be terminated when any of the following criteria is met: (1) AEs related to study drug of CTCAE Grade 2 and above occur in ≥1/2 subjects of one dose group; (2) AEs related to study drug of CTCAE Grade 3 and above occur in ≥1/3 subjects of one dose group; (3) SAE related to study drug occurs in at least one subject; (4) ALT or AST > 3×ULN and total bilirubin (TBiL) >2×ULN, non-biliary elevation (usually alkaline phosphatase <2×ULN) occurs in a dose group, and could not be explained by other diseases; (5) urine crystallization is observed in at least two subjects in a certain dose group, with glomerular filtration rate <60 mL/min/1.73 m^2^, and could not be explained by other reasons; (6) no need to continue the trial based on the obtained trial data.

### Biological sample collection

Approximately 3 mL blood samples were collected at the scheduled timepoint for analysis. In 25 mg group of single ascending-dose study, blood samples were collected at the following 15 time points: 0 (pre-dose), 0.25, 0.5, 1, 1.5, 2, 3, 4, 5, 6, 8, 12, 24, 36 and 48 h post dose. Afterward, the time points were slightly adjusted depending on the pharmacokinetic profile of the 25-mg group. In other dose groups of single ascending-dose study, as well as food-effect study, blood samples were collected at the following time points: 0 (pre-dose), 10 min, 20 min, 30 min, 45 min, 1 h, 1.5 h, 2 h, 3 h, 4 h, 6 h, 8 h, 12 h, 24 h, and 48 h post dose.

In multiple ascending-dose study, blood samples were collected at the following time points: 0 (pre-dose), 10 min, 20 min, 30 min, 45 min, 1 h, 1.5 h, 2 h, 3 h, 4 h, 6 h, 8 h, and 12 h post first dose at day 1; each pre-dose at day 5; 0 (pre-dose), 10 min, 20 min, 30 min, 45 min, 1 h, 1.5 h, 2 h, 3 h, 4 h, 6 h, 8 h, 12 h, 24 h and 48 h post dose at day 6.

The collected blood samples were separated by centrifuging at 4 °C, 1500 × *g* for 10 min, then were divided into two centrifuge tubes (at least 0.6 mL plasma each), frozen, and stored under −80 °C until analysis.

Exploratory mass balance evaluation was conducted in 400 mg dose group of single ascending-dose study. Random urine sample within 24 h to 0 h prior to administration on day 1, and total excreted urine volumes in the time intervals of 0–6, 6–12, 12–24, 24–48, and 48–72 h after administration were collected respectively. Random fecal samples within 24 h to 0 h prior to the administration on day 1, and within 0–72 h after administration were collected, respectively.

### Bioanalytical procedures

A simple, precise and accurate LC-MS/MS method was developed and validated to determine the concentration of VV116 and its metabolite 116-N1 in human plasma.

For VV116, VV116-D4 was chosen as the internal standard. Under the calibration range (2–2000 ng/mL), the regression coefficient was 0.9998. The intra-run precision ranged from 1.1% to 6.6%, and the inter-run precision from 4.6% to 9.6%. The accuracies were 85.6%–102.3%.

For the metabolite 116-N1, 116-N1-D4 was chosen as the internal standard. The calibration curves were linear over 10–10,000 ng/mL, of which the regression coefficient was 0.9998. The intra-run precision ranged from 2.2% to 12.4%, and the inter-run precision from 2.5% to 8.2%. The accuracies were 90.5%–100.1%.

### Pharmacokinetic assessments

PK parameters were calculated using a non-compartmental (NCA) model by WinNonlin Software version 8.3.1 (Pharsight, Cary, NC, USA). Main PK parameters included maximum observed plasma concentration (*C*_max_), area under the plasma concentration–time curve (AUC), AUC from time zero (pre-dose) to the time of the last measurable concentration (AUC_0-t_), AUC from time zero (pre-dose) extrapolated to infinity (AUC_0-∞_), area under the concentration–time curve during a dosing interval (AUC_TAU), time of maximum plasma concentration (*T*_max_), terminal plasma elimination half-life (*t*_1/2_), apparent volume of distribution (*V*_d_/F), apparent clearance (CL/F), terminal phase elimination rate constant (*K*_e_), mean residence time (MRT), cumulative excretion (*A*_e_), and fractional excretion (*F*_e_). AUC_0-t_ and AUC_0-∞_ were calculated using a Linear Up Log Down rule method. *T*_max_ and *C*_max_ were based on the actual measured values. In multiple ascending-dose study, accumulation ratio after repeated dosing (R_ac_) was also analyzed.

### Statistical analyses

Statistical analysis was performed using SAS Software version 9.4 (SAS Institute, Cary, NC, USA). Descriptive statistics were expressed as arithmetic mean, standard deviation, coefficient of variation, median, maximum, minimum, and geometric mean of each dose group. Frequency and percentage were calculated to summarize categorical variables.

In the single ascending-dose study, the dose-exposure relationship was assessed using confidence interval criteria. The linear regression was carried out. The regression equation was expressed as ln(PK) = α + β×ln(Dose) where logarithmic transformation was used for PK parameters and doses. The 90% confidence interval of the estimated slope (β) was calculated. PK parameters were considered to be linearly associated to dose, in the condition that the 90% confidence interval of β was within the judgment interval.

In the food-effect study, PK parameters including AUC_0-t_, AUC_0-∞_, and *C*_max_ under different dietary conditions were analyzed. The mean, mean difference and 90% confidence interval of logarithm transformed PK parameter were estimated using generalized linear mixed model, where the dietary condition, sequence, and period were included as a fixed effect, while the subject was included as a random effect. The pairwise comparisons (high-fat meal vs. fasting, standard meal vs. fasting, high-fat meal vs. standard meal) were carried out for each PK parameter. Food intake was considered to have no effect on the bioavailability of VV116, in the condition that the 90% confidence interval of the geometric mean ratio of AUC_0-t_, AUC_0-∞_, and *C*_max_ under fed condition was within 80%–125% of that under fasting condition.

## Results

### Demographic profile

**Fig. 2:**
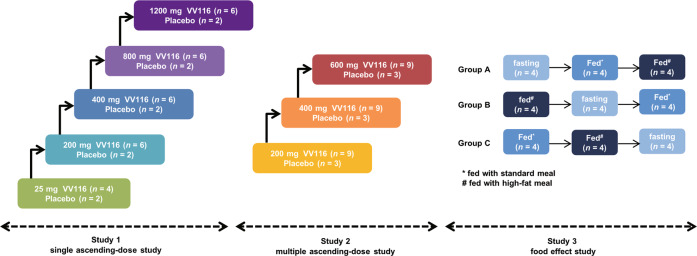
The scheme of study design. Three studies (Study 1, Study 2, and Study 3) were launched sequentially.

A total of 86 eligible subjects (38 in single ascending-dose study, 36 in multiple ascending-dose study, and 12 in food-effect study) were enrolled and completed the study. No subject was discontinued from the study. Subject distribution is displayed in Fig. 2. The demographic characteristics of all enrolled subjects are summarized in Table 1.

**Table 1:** Demographic profile of enrolled subjects.

	Single ascending-dose study						Multiple ascending-dose study				Food-effect study		
	25 mg (*n* = 4)	200 mg (*n* = 6)	400 mg (*n* = 6)	800 mg (*n* = 6)	1200 mg (*n* = 6)	Placebo (*n* = 10)	200 mg (*n* = 9)	400 mg (*n* = 9)	600 mg (*n* = 9)	Placebo (*n* = 9)	Group A^*^ (*n* = 4)	Group B^*^ (*n* = 4)	Group C^*^ (*n* = 4)
Age, year	27.3 (7.18)	31.3 (4.89)	29.7 (5.65)	27.2 (5.12)	31.2 (7.73)	29.2 (6.80)	28.0 (3.46)	29.2 (6.34)	29.0 (6.06)	30.3 (6.73)	26.5 (4.43)	27.8 (3.83)	29.0 (7.26)
Gender													
Male	4 (100)	6 (100)	6 (100)	5 (83.3)	6 (100)	8 (80.0)	8 (88.9)	9 (100)	9 (100)	8 (88.9)	3 (75.0)	4 (100)	4 (100)
Female	0	0	0	1 (16.7)	0	2 (20.0)	1 (11.1)	0	0	1 (11.1)	1 (25.0)	0	0
Height, cm	171.0 (6.48)	170.6 (4.33)	168.1 (2.69)	165.2 (9.07)	167.4 (5.34)	166.7 (8.62)	168.8 (7.55)	167.0 (5.08)	165.5 (3.95)	164.9 (3.92)	163.4 (4.79)	169.5 (4.98)	166.9 (4.25)
Weight, kg	65.6 (9.79)	60.3 (5.50)	62.7 (4.18)	65.0 (11.38)	63.4 (8.52)	63.8 (9.61)	64.3 (6.42)	63.9 (6.96)	60.9 (5.55)	60.5 (5.70)	57.6 (6.21)	64.6 (7.11)	63.0 (5.42)
BMI, kg/m^2^	22.3 (1.73)	20.7 (1.07)	22.2 (1.16)	23.7 (2.45)	22.5 (1.77)	22.8 (1.51)	22.6 (1.89)	22.9 (1.75)	22.2 (1.61)	22.2 (2.04)	21.6 (1.36)	22.4 (1.12)	22.6 (1.71)

*BMI* body mass index.

Note**:** Data are expressed as mean (SD), except for gender, which is shown as *n* (%).

^*^Group A, B, and C experienced three treatment periods, respectively.

Group A: fasting→standard meal→high-fat meal.

Group B: high-fat meal→fasting→standard meal.

Group C: standard meal→high-fat meal→fasting.

### Pharmacokinetic properties

#### Study 1: single ascending-dose study

VV116 was hydrolyzed rapidly to its metabolite 116-N1 after oral administration. The prototype drug was not detected (lower limit of quantitation was 2 ng/mL) in plasma, while its metabolite 116-N1 was detected, and calculated for the PK parameters in the three studies.

**Fig. 3:**
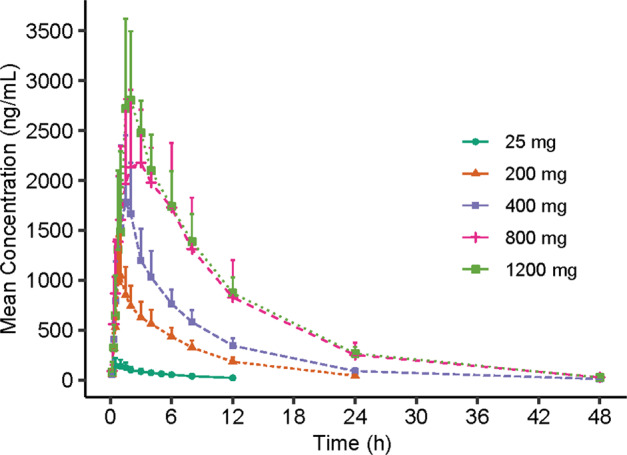
The mean plasma 116-N1 concentration-time curves in each dose group after a single dose of VV116. Mean (SD) of plasma 116-N1 concentration is displayed in this figure.

The main PK parameters of 116-N1 in each dose group after a single dose of VV116 are summarized in Table 2. The mean plasma 116-N1 concentration–time curves are demonstrated in Fig. 3.

**Table 2:** The main PK parameters of 116-N1 in each dose group after a single dose of VV116.

Pharmacokinetic parameters	25 mg (*n* = 4)	200 mg (*n* = 6)	400 mg (*n* = 6)	800 mg (*n* = 6)	1200 mg (*n* = 6)
AUC_0-t_ (h·ng/mL)	744 (244)	6631 (1603)	12759 (2747)	25886 (5904)	28057 (5145)
AUC_0-∞_ (h·ng/mL)	904 (301)	6986 (1683)	13064 (2727)	26233 (5897)	28325 (5272)
*C*_max_ (ng/mL)	165 (74.0)	1096 (412)	1898 (701)	2796 (225)	3086 (778)
*T*_max_ (h)	1.00 (0.50, 1.50)	1.00 (0.75, 1.50)	1.50 (1.00, 2.00)	2.50 (1.50, 6.00)	2.00 (1.50, 3.00)
*t*_1/2_ (h)	4.80 (0.492)	5.48 (0.430)	6.15 (1.08)	6.75 (1.36)	6.95 (0.659)
*K*_e_ (1/h)	0.145 (0.0135)	0.127 (0.0111)	0.115 (0.0200)	0.107 (0.0273)	0.100 (0.00886)
*V*_d_/F (L)	214 (94.3)	240 (73.4)	285 (97.5)	306 (79.6)	432 (53.9)
CL/F (L/h)	31.4 (15.3)	30.4 (8.71)	31.9 (7.29)	31.7 (6.70)	43.6 (8.26)
MRT (h)	6.97 (0.156)	8.15 (0.829)	8.78 (1.96)	10.5 (2.00)	10.6 (0.825)
Urine (116-N1)					
*A*_e__0-72_ (mg)			107 (25.3)		
*F*_e__0-72_ (%)^*^			53.6 (12.6)		
Feces (116-N1 + VV116^**^)					
*A*_e__0-72_ (mg)			10.6 (10.4)		
*F*_e__0-72_ (%)^*^			5.25 (5.15)		

*AUC*_*0-t*_ area under the concentration–time curve from time zero to the time of the last measurable concentration, *AUC*_*0-∞*_ area under the concentration–time curve from time zero to infinity, *C*_*max*_ maximum observed plasma concentration, *T*_*max*_ time to maximum plasma concentration, *t*_*1/2*_ terminal elimination half-life, *Ke* elimination rate constant, *Vd/F* apparent distribution volume, *CL/F* clearance rate, *MRT* mean retention time, *Ae* cumulative excretion, *Fe* fractional excretion.

Note: Data are expressed as mean (SD), except for *T*_max_, which is shown as median (min, max).

*VV116 hydrobromide (MW: 583.46) was calculated for VV116. VV116 400 mg was converted to 116-N1 (MW: 292.27) 200.37 mg in the evaluation of *F*_e__0-72_ for 116-N1.

**The concentration of VV116 was detected in feces, though it was very low.

The main PK exposure parameters *C*_max_ and AUC were dose-dependent. The mean ± SD of *C*_max_ was 165 ± 74.0, 1096 ± 412, 1898 ± 701, 2796 ± 225 and 3086 ± 778 ng/mL for 25 mg, 200 mg, 400 mg, 800 mg and 1200 mg dose groups, respectively. AUC_0-t_ was 744 ± 244, 6631 ± 1603, 12759 ± 2747, 25886 ± 5904 and 28057 ± 5145 h·ng/mL, while AUC_0-∞_ was 904 ± 301, 6986 ± 1683, 13064 ± 2727, 26233 ± 5897 and 28325 ± 5272 h·ng/mL for the above five dose groups. Confidence interval (CI) criteria were used to assess the dose linear relationship. Table 3 demonstrates the dose proportionality analysis. The slope estimate (β) and its 90% CI for *C*_max_, AUC_0-t_, and AUC_0-∞_ was 0.85 (0.73–0.97), 1.03 (0.95–1.11), and 0.98 (0.90–1.06) for 25–800 mg dose interval, which was not completely within the judgment interval (0.94–1.06). Even though, we could draw a conclusion that AUC parameters and *C*_max_ increased in an approximately dose-proportional manner in the dose range of 25–800 mg. However, *C*_max_, AUC_0-t_, and AUC_0-∞_ did not show significant change with dose escalation from 800 to 1200 mg. The median of *T*_max_ was within 1.00–2.50 h, and the mean *t*_1/2_ was within 4.80–6.95 h (Table 2).

**Table 3:** The dose proportionality analysis for *C*_max_ and AUC of plasma 116-N1 in a single ascending-dose study.

PK parameters	Dose interval	Slope estimate (β) (90% CI)	Judgment interval
*C*_max_ (ng/mL)	25–1200 mg	0.79 (0.69, 0.89)	0.94–1.06
AUC_0-t_ (h·ng/mL)	25–1200 mg	0.97 (0.90, 1.04)	0.94–1.06
AUC_0-∞_ (h·ng/mL)	25–1200 mg	0.92 (0.85, 0.99)	0.94–1.06
*C*_max_ (ng/mL)	25–800 mg	0.85 (0.73, 0.97)	0.94–1.06
AUC_0-t_ (h·ng/mL)	25–800 mg	1.03 (0.95, 1.11)	0.94–1.06
AUC_0-∞_ (h·ng/mL)	25–800 mg	0.98 (0.90, 1.06)	0.94–1.06

#### Study 2: multiple ascending-dose study

**Fig. 4:**
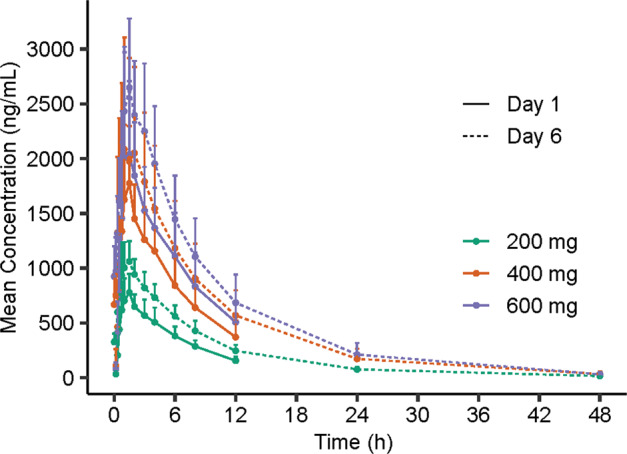
The mean plasma 116-N1 concentration-time curves at Day 1 and Day 6 in multiple ascending-dose study. Mean (SD) of plasma 116-N1 concentration is displayed in this figure.

The main PK parameters of 116-N1 at day 1 and day 6 after multiple doses of VV116 are presented in Table 4. The mean plasma 116-N1 concentration–time curves at day 1 and day 6 of 200, 400, and 600 mg dose groups are demonstrated in Fig. 4. Table 5 displays the trough concentrations of 116-N1 at day 5 and day 6.

**Table 4:** The main PK parameters of 116-N1 at day 1 and day 6 in multiple ascending-dose study.

Pharmacokinetic parameters	200 mg (*n* = 9)		400 mg (*n* = 9)		600 mg (*n* = 9)	
	Day 1	Day 6	Day 1	Day 6	Day 1	Day 6
AUC_0-t_ (h·ng/mL)^*^	4610 (864)	9384 (1880)	10351 (2843)	20774 (8321)	12871 (3309)	25077 (7100)
AUC_0-∞_ (h·ng/mL)	5690 (981)	9664 (1809)	13071 (4244)	21195 (8611)	16581 (5175)	25448 (7329)
*C*_max_ (ng/mL)	858 (186)	1131 (231)	1968 (670)	2304 (851)	2418 (708)	2842 (617)
*T*_max_ (h)	1.50 (0.750, 1.50)	1.00 (0.750, 1.50)	1.50 (0.750, 1.50)	1.00 (0.330, 3.00)	1.50 (1.00, 6.00)	1.50 (1.00, 3.00)
*t*_1/2_ (h)	4.72 (1.02)	7.56 (1.59)	4.88 (0.718)	8.12 (0.903)	5.41 (0.808)	7.85 (0.692)
CL/F (L/h)	36.2 (6.84)	21.4 (4.28)	33.3 (9.91)	21.7 (8.20)	39.7 (13.9)	25.2 (6.83)
MRT (h)	7.42 (1.24)	9.91 (1.71)	7.56 (1.11)	10.6 (1.28)	8.20 (1.38)	10.1 (1.38)
AUC_TAU^**^		6959 (1148)		14745 (5419)		17966 (4205)
R_ac__*C*_max_ (%)		1.34 (0.206)		1.18 (0.219)		1.24 (0.336)
R_ac__AUC (%)^***^		1.53 (0.235)		1.41 (0.239)		1.42 (0.175)

*AUC*_*0-t*_ area under the concentration–time curve from time zero to the time of the last measurable concentration, *AUC*_*0-∞*_ area under the concentration–time curve from time zero to infinity, *C*_*max*_ maximum observed plasma concentration, *T*_*max*_ time to maximum plasma concentration, *t*_*1/2*_ terminal elimination half-life, *CL/F* clearance rate, *MRT* mean retention time, *AUC_TAU* area under the concentration–time curve during a dosing interval, *R*_*ac*_ accumulation ratio at steady state.

Note**:** Data are expressed as mean (SD), except for *T*_max_, which is shown as median (min, max).

*AUC_0-t_: the last timepoint was 12 h on day 1 and 48 h on day 6.

**AUC_TAU: AUC from time 0 to 12 h.

***R_ac__AUC was calculated based on AUC_0-t_ on day 1 and AUC_TAU on day 6.

**Table 5:** The trough concentrations of 116-N1 at day 5 and day 6 in multiple ascending-dose study.

Concentration (ng/mL)	200 mg (*n* = 9)	400 mg (*n* = 9)	600 mg (*n* = 9)
Pre-dose of 1st dosing at day 5	345 (88.2)	766 (310)	1011 (311)
Pre-dose of 2nd dosing at day 5	242 (56.9)	559 (242)	721 (272)
Pre-dose at day 6	326 (75.7)	669 (262)	924 (277)

Note**:** Data are expressed as mean (SD).

The mean *t*_1/2_ at day 6 was longer than that at day 1 no matter in which dose group (4.72 h vs. 7.56 h in 200 mg group, 4.88 h vs. 8.12 h in 400 mg group, 5.41 h vs. 7.85 h in 600 mg group). As for drug exposure, *C*_max_, AUC_0-t,_ and AUC_0-∞_ increased after repeated dosing. The accumulation ratios (R_ac_) of *C*_max_ were 1.34, 1.18, and 1.24, and R_ac_ of AUC were 1.53, 1.41, and 1.42 for 200, 400, and 600 mg dose groups, respectively, which showed a slight accumulation upon repeated dosing of VV116.

#### Study 3: food-effect study

**Fig. 5:**
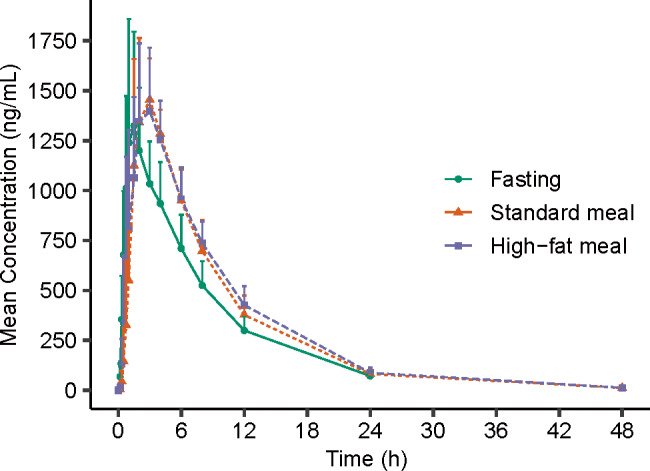
The mean plasma 116-N1 concentration-time curves under fasting and fed conditions. Mean (SD) of plasma 116-N1 concentration is displayed in this figure.

The key PK parameters of 116-N1 after a single oral dose of 400 mg VV116 under different diet conditions are listed in Table 6, and the corresponding mean plasma drug concentration–time curves are displayed in Fig. 5.

**Table 6:** The main PK parameters of 116-N1 under fasting and fed conditions after a single oral dose of 400 mg VV116.

Pharmacokinetic parameters	Fasting (*n* = 12)	Standard meal (*n* = 12)	High-fat meal (*n* = 12)
AUC_0-t_ (h·ng/mL)	10443 (2031)	12405 (1941)	13107 (2042)
AUC_0-∞_ (h·ng/mL)	10962 (2020)	12889 (1947)	13600 (2126)
*C*_max_ (ng/mL)	1523 (434)	1583 (273)	1602 (270)
*T*_max_ (h)	1.50 (1.00, 4.00)	3.00 (1.50, 6.00)	2.50 (1.00, 6.00)
*t*_1/2_ (h)	5.66 (0.700)	5.31 (0.651)	5.47 (0.787)
*V*_d_/F (L)	308 (72.2)	240 (31.8)	234 (22.6)
CL/F (L/h)	37.7 (7.23)	31.7 (4.89)	30.1 (4.62)
FE_ *C*_max_ (%)		106.60 (93.40, 121.67)^*^	107.92 (94.56, 123.18)^**^
FE_ AUC_0-t_ (%)		119.52 (114.50, 124.76)^*^	126.32 (121.01, 131.85)^**^
FE_ AUC_0-∞_(%)		118.21 (113.53, 123.08)^*^	124.67 (119.74, 129.81)^**^

*AUC*_*0-t*_ area under the concentration–time curve from time zero to the time of the last measurable concentration, *AUC*_*0-∞*_ area under the concentration–time curve from time zero to infinity, *C*_*max*_ maximum observed plasma concentration, *T*_*max*_ time to maximum plasma concentration, *t*_*1/2*_ terminal elimination half-life, *Vd/F* apparent distribution volume, *CL/F* clearance rate, *FE* food effect.

Note: Data are expressed as Mean (SD), except for *T*_max_, which is shown as median (min, max).

^*^Geometric mean ratios (90% CIs) of *C*_max_, AUC_0-t_ and AUC_0-n_ between standard meal and fasting.

^**^Geometric mean ratios (90% CIs) of *C*_max_, AUC_0-t_ and AUC_0-∞_ between high-fat meal and fasting.

The geometric mean ratios (GMR) and their 90% CIs of *C*_max_, AUC_0-t_, and AUC_0-∞_ between standard meal and fasting were 106.60% (93.40%–121.67%), 119.52% (114.50%–124.76%), and 118.21% (113.53%–123.08%), respectively, which was within the equivalent range 80%–125%. The GMR (90% CIs) of *C*_max_, AUC_0-t_, and AUC_0-∞_ between high-fat meal and fasting were 107.92% (94.56%–123.18%), 126.32% (121.01%–131.85%), and 124.67% (119.74%–129.81%), respectively. AUC_0-t_ and AUC_0-∞_ increased by 26.32% and 24.67% with a high-fat meal compared to the fasting condition.

The median *T*_max_ of 116-N1 under fasting condition, fed condition with a standard meal, fed condition with a high-fat meal was 1.5, 3.0, and 2.5 h, respectively. We figured out that VV116 administration under fed condition could prolong the time to the peak as compared with the fasting condition, but had little effect on the system exposure of the study drug.

### Safety

No deaths, serious adverse event (SAE), AEs of Grade 3 or above, or AEs leading to drug discontinuation/interruption were reported throughout the three studies. All AEs were recovered without any treatment or intervention.

#### Study 1: single ascending-dose study

The number (incidence) of subjects experiencing AEs for 25, 200, 400, 800, and 1200 mg dose group and placebo group was 2 (50%), 3 (50%), 3 (50%), 3 (50%), 0 (0), and 5 (50%), respectively (Table 7). No relation with dose was observed for the AE occurrence. The incidence of AE for subjects administered VV116 in total was lower than those administered placebo (39.3% vs. 50%) in a single ascending-dose study. The severity of AEs was CTCAE Grade 1 with the exception of one case of Grade 2 neutropenia. The dose-escalation termination criteria were not met during dose escalation. The most common drug-related AEs were sinus bradycardia, shortened electrocardiogram PR, and increased blood bilirubin.

**Table 7:** Summary of adverse events for enrolled subjects in single ascending-dose study.

Adverse events, *n* (%)	25 mg (*n* = 4)	200 mg (*n* = 6)	400 mg (*n* = 6)	800 mg (*n* = 6)	1200 mg (*n* = 6)	Total VV116 (*n* = 28)	Placebo (*n* = 10)
Overall	2 (50.0)	3 (50.0)	3 (50.0)	3 (50.0)	0	11 (39.3)	5 (50.0)
VV116/placebo related	2 (50.0)	3 (50.0)	3 (50.0)	3 (50.0)	0	11 (39.3)	4 (40.0)
Severity							
Grade 1	2 (50.0)	3 (50.0)	2 (33.3)	3 (50.0)	0	10 (35.7)	5 (50.0)
Grade 2	0	0	1 (16.7)	0	0	1 (3.6)	0
Grade 3	0	0	0	0	0	0	0
Grade 4	0	0	0	0	0	0	0
Grade 5	0	0	0	0	0	0	0
VV116/placebo-related AEs reported by more than 1 subject							
Electrocardiogram PR shortened	0	0	2 (33.3)	1 (16.7)	0	3 (10.7)	1 (10.0)
Blood bilirubin increased	1 (25.0)	1 (16.7)	0	0	0	2 (7.1)	0
Blood triglycerides increased	0	0	0	1 (16.7)	0	1 (3.6)	1 (10.0)
Blood uric acid increased	0	0	0	1 (16.7)	0	1 (3.6)	1 (10.0)
Red blood cells urine positive	0	1 (16.7)	0	0	0	1 (3.6)	1 (10.0)
White blood cells urine positive	0	1 (16.7)	0	0	0	1 (3.6)	2 (20.0)
Sinus bradycardia	1 (25.0)	1 (16.7)	2 (33.3)	0	0	4 (14.3)	0

Note: *n* (%), number (incidence) of subjects with an adverse event.

#### Study 2: multiple ascending-dose study

The number (incidence) of subjects experiencing AEs for 200 mg, 400 mg, 600 mg dose group and placebo group was 3 (33.3%), 5 (55.6%), 6 (66.7%), and 5 (55.6%), respectively (Table 8). The incidence of AE for subjects administered VV116 in total was comparable with those administered placeboes (51.9% vs. 55.6%). AE occurrence was detected to be related to dose. Besides one subject in the placebo group experienced three cases of Grade 2 nausea, the severity of AEs was generally mild (CTCAE Grade 1). The most common drug-related AEs were increased blood uric acid, dry mouth, presence of crystal urine, and nausea. Three cases had increased transaminases (increased alanine aminotransferase, increased aspartate aminotransferase, and increased gamma-glutamyltransferase) with Grade 1 observed in two subjects of 400 mg dose group. Transaminase increase was transient, and recovered spontaneously.

**Table 8:** Summary of adverse events for enrolled subjects in multiple ascending-dose study.

Adverse events, *n* (%)	200 mg (*n* = 9)	400 mg (*n* = 9)	600 mg (*n* = 9)	Total VV116 (*n* = 27)	Placebo (*n* = 9)
Overall	3 (33.3)	5 (55.6)	6 (66.7)	14 (51.9)	5 (55.6)
VV116/placebo related	3 (33.3)	5 (55.6)	6 (66.7)	14 (51.9)	5 (55.6)
Severity					
Grade 1	3 (33.3)	5 (55.6)	6 (66.7)	14 (51.9)	4 (44.4)
Grade 2	0	0	0	0	1 (11.1)
Grade 3	0	0	0	0	0
Grade 4	0	0	0	0	0
Grade 5	0	0	0	0	0
VV116/placebo-related AEs reported by more than 1 subject					
Blood uric acid increased	0	2 (22.2)	2 (22.2)	4 (14.8)	0
Crystal urine present	0	1 (11.1)	1 (11.1)	2 (7.4)	1 (11.1)
Dry mouth	2 (22.2)	0	1 (11.1)	3 (11.1)	2 (22.2)
Nausea	0	0	2 (22.2)	2 (7.4)	2 (22.2)
Supraventricular extrasystoles	0	0	1 (11.1)	1 (3.7)	1 (11.1)

Note**:** *n* (%), number (incidence) of subjects with an adverse event.

#### Study 3: food-effect study

The number (incidence) of subjects experiencing AEs under fasting condition, fed condition with a standard meal, fed condition with a high-fat meal was 0 (0), 2 (16.7%), and 4 (33.3%). Two subjects under fed condition with a standard meal were observed atrioventricular block with first degree, while four subjects under the fed condition with a high-fat meal experienced positive results in urine bacterial test, presence of crystal urine, increase in blood pressure, and atrioventricular block with first degree. All AEs were CTCAE Grade 1 in severity.

#### Other safety assessments

Only one subject in 400 mg dose group of Study 3 experienced mild increase in transient blood thyroid-stimulating hormone, which recovered spontaneously without any treatment. No clinically significant abnormality was discovered in sex hormone test, ophthalmological examination, and thyroid B ultrasound test.

## Discussion

VV116 is a prodrug of nucleoside analog, intended for the treatment of COVID-19. RDV is the first FDA-approved drug for the treatment of COVID-19, which is also a nucleoside analog. Compared with RDV, VV116 exhibits better in vitro antiviral activity and selectivity [14]. In addition, VV116 could be administered orally and has favorable oral bioavailability, that is more convenient for COVID-19 patients than intravenous administration of RDV.

VV116 was hydrolyzed rapidly to its metabolite 116-N1 after oral administration. 116-N1, instead of the prototype drug VV116, was detected in plasma, and calculated for the PK parameters. Peak plasma 116-N1 concentration reached quickly after oral administration (median *T*_max_ 1.00–2.50 h). In the single ascending-dose study, AUC and *C*_max_ increased in an approximately dose-proportional manner in the dose range of 25–800 mg. However, the parameters did not show significant change with dose escalation from 800 to 1200 mg (AUC_0-t_: 25886 vs. 28057 h·ng/mL; *C*_max_: 2796 vs. 3086 ng/mL), indicating the probability of drug absorption saturation. Drug solubility is an important factor affecting the drug absorption and maximum drug absorption occurs when the drug has maximum concentration (saturation solubility) at the site of absorption. It was suspected that limited solubility of VV116 might be the reason for drug absorption saturation. The fractional excretion of 116-N1 in urine was 53.6% in 0–72 h after administration, while that of 116-N1 and VV116 in feces was 5.25%, which indicated that VV116 was principally excreted through kidney in the form of metabolite 116-N1.

The mean *t*_1/2_ of VV116 was 4.80–6.95 h in the single ascending-dose study, suggesting twice-daily dosing in the clinical treatment. Thereby, continuous twice-daily dosing (12 h apart) for 5.5 days (days 1–6) was adopted in the multiple ascending-dose study. The accumulation ratio of AUC parameters and *C*_max_ indicated a slight accumulation of VV116 after continuous dosing. The trough concentrations of 116-N1 following multiple administration of 200 mg at day 5 and day 6 were within 242–345 ng/mL (Table 5), which were above the EC_90_ (186.5 ng/mL) of 116-N1 against the omicron variant in a preclinical anti-SARS-CoV-2 assay. Therefore, the dosage regimen of 200 mg BID and above can continuously maintain the effective antiviral concentration, and is recommended for subsequent clinical studies in patients with COVID-19.

The median *T*_max_ under fasting, standard meal and high-fat meal condition was 1.50, 3.00, and 2.50 h, respectively, indicating that fed condition could prolong the time to the peak. Compared with fasting condition, the GMR (90% CIs) of *C*_max_ under fed condition with both standard meal and high-fat meal was within the equivalent range 80%–125%; the GMR (90% CIs) of AUC for standard meal was also within the range 80%–125%, however for high-fat meal, AUC_0-t_ and AUC_0-∞_ slightly increased by 26.32% and 24.67%, respectively. Since food intake has no effect on *C*_max_ of VV116, while high-fat meal slightly increases AUC, it is recommended that VV116 could be taken under fasting condition or fed condition with regular meal in the treatment of COVID-19.

In the single ascending-dose study, there was no apparent dose-related trend, with a greater proportion of subjects reporting AEs following administration of placebo (50.0%) than following administration of VV116 (39.3%). The severity of AEs was CTCAE Grade 1 with the exception for one case of Grade 2 neutropenia. In the multiple ascending-dose study, the incidence of AEs in the VV116 group was comparable with that in the placebo group (51.9% vs. 55.6%). AE occurrence was slightly dose-related. Only 1 subject in 400 mg dose group reported one case of increased alanine aminotransferase and increased aspartate aminotransferase, respectively. All AEs in subjects administered VV116 were Grade 1 in severity, and were recovered without any treatment. No serious adverse event occurred throughout the study, and no subject withdrew from the study due to AE. In the preclinical animal toxicology study, it was discovered that VV116 might have toxicity on eyes, thyroid, and gonads. In our studies, ophthalmology examination, thyroid function, thyroid B ultrasound, and sex hormone tests were performed on healthy subjects before and after VV116 administration. No obvious toxicity was observed in the above organs. Overall, VV116 demonstrated satisfactory safety profiles in healthy subjects throughout the three studies.

Hepatotoxicity is the primary adverse drug reaction (ADR) of RDV, manifested as transaminase elevation. In phase I clinical study (Study GS-US-399-5505), subjects received one loading dose of 200 mg RDV followed by 100 mg for up to 9 days, transient ALT elevation of Grade 1 or 2 was observed in 9 of 20 subjects (45%) [16]. Transaminase elevation has also been reported as the most frequent ADR in patients with COVID-19 who received RDV [17, 18]. In the multiple ascending-dose study of VV116, only 1 of 27 subjects (3.7%) experienced transient ALT elevation of Grade 1, which recovered spontaneously after VV116 termination. This can be explained by the high targeting capability of RDV to the liver and its liver/blood concentration ratio is about 21 times that of VV116. The liver/blood concentration ratio of RDV (calculated by equivalents ^14^C-GS-5734) after a single intravenous administration of 10 mg/kg [^14^C]RDV at 4 h was 57.8 [19], while the ratio of the VV116 (calculated by major metabolite 116-N1) after a single oral dose of 30 mg/kg VV116 to rat at 2 h was only 2.8. Despite the lower risk of hepatotoxicity of VV116 compared to RDV, monitoring for the hepatic function will continue in the subsequent phase II study of VV116 in COVID-19 patients.

## Conclusions

VV116 exhibited satisfactory safety and tolerability in healthy subjects. Peak plasma drug concentration of 116-N1 reached quickly after oral administration of VV116 (median *T*_max_ 1.00–2.50 h). AUC and *C*_max_ increased in an approximately dose-proportional manner in the dose range of 25–800 mg, while drug absorption saturation was probably achieved at the dose of 800 mg. Standard meal had no effect on *C*_max_ and AUC of VV116. Effective antiviral concentration was achieved at dose levels between 200 and 600 mg BID following multiple administration.

In conclusion, the safety data and PK profile from these studies support the continued investigation of VV116 in patients with COVID-19.
